# Long non-coding RNA MAPKAPK5-AS1/PLAGL2/HIF-1α signaling loop promotes hepatocellular carcinoma progression

**DOI:** 10.1186/s13046-021-01868-z

**Published:** 2021-02-17

**Authors:** Liang Wang, Liankang Sun, Runkun Liu, Huanye Mo, Yongshen Niu, Tianxiang Chen, Yufeng Wang, Shaoshan Han, Kangsheng Tu, Qingguang Liu

**Affiliations:** Department of Hepatobiliary Surgery, The First Affiliated Hospital of Xi’an Jiaotong University, 277 Yanta West Road, Xi’an, 710061 China

**Keywords:** MAPKAPK5-AS1, miR-154-5p, PLAGL2, Hepatocellular carcinoma progression, Hypoxia

## Abstract

**Background:**

Long non-coding RNAs (lncRNAs) are widely involved in human cancers’ progression by regulating tumor cells’ various malignant behaviors. MAPKAPK5-AS1 has been recognized as an oncogene in colorectal cancer. However, the biological role of MAPKAPK5-AS1 in hepatocellular carcinoma (HCC) has not been explored.

**Methods:**

Quantitative real-time PCR was performed to detect the level of MAPKAPK5-AS1 in HCC tissues and cell lines. The effects of MAPKAPK5-AS1 on tumor growth and metastasis were assessed via in vitro experiments, including MTT, colony formation, EdU, flow cytometry, transwell assays, and nude mice models. The western blotting analysis was carried out to determine epithelial-mesenchymal transition (EMT) markers and AKT signaling. The interaction between MAPKAPK5-AS1, miR-154-5p, and PLAGL2 were explored by luciferase reporter assay and RNA immunoprecipitation. The regulatory effect of HIF-1α on MAPKAPK5-AS1 was evaluated by chromatin immunoprecipitation.

**Results:**

MAPKAPK5-AS1 expression was significantly elevated in HCC, and its overexpression associated with malignant clinical features and reduced survival. Functionally, MAPKAPK5-AS1 knockdown repressed the proliferation, mobility, and EMT of HCC cells and induced apoptosis. Ectopic expression of MAPKAPK5-AS1 contributed to HCC cell proliferation and invasion in vitro. Furthermore, MAPKAPK5-AS1 silencing suppressed, while MAPKAPK5-AS1 overexpression enhanced HCC growth and lung metastasis in vivo. Mechanistically, MAPKAPK5-AS1 upregulated PLAG1 like zinc finger 2 (PLAGL2) expression by acting as an endogenous competing RNA (ceRNA) to sponge miR-154-5p, thereby activating EGFR/AKT signaling. Importantly, rescue experiments demonstrated that the miR-154-5p/PLAGL2 axis mediated the function of MAPKAPK5-AS1 in HCC cells. Interestingly, we found that hypoxia-inducible factor 1α (HIF-1α), a transcript factor, could directly bind to the promoter to activate MAPKAPK5-AS1 transcription. MAPKAPK5-AS1 regulated HIF-1α expression through PLAGL2 to form a hypoxia-mediated MAPKAPK5-AS1/PLAGL2/HIF-1α signaling loop in HCC.

**Conclusions:**

Our results reveal a MAPKAPK5-AS1/PLAGL2/HIF-1α signaling loop in HCC progression and suggest that MAPKAPK5-AS1 could be a potential novel therapeutic target of HCC.

**Supplementary Information:**

The online version contains supplementary material available at 10.1186/s13046-021-01868-z.

## Background

Hepatocellular carcinoma (HCC), as the most common type of liver cancer, is the third leading cause of tumor-related deaths worldwide [1]. Despite advances made in HCC treatment, surgical resection is still the main treatment method. However, the prognosis of HCC patients is still not satisfactory, and the reason is mainly due to metastasis and recurrence after surgical removal of the tumor [2]. The exact mechanism of tumor occurrence and metastasis remain largely unclear. Therefore, it is very urgent and essential to clarify tumorigenesis and metastasis’s molecular mechanism and develop new treatments for HCC.

In recent years, numerous long non-coding RNAs (lncRNAs) have been discovered and proved by many studies to be involved in the progression of various tumors, including HCC [3–6]. For example, Zhe Li et al. reported that LINC00624 enhances liver cancer progression by disrupting the histone deacetylase 6 (HDAC6)- tripartite motif containing 28 (TRIM28) - zinc finger potein 354C (ZNF354C) corepressor complex [6]. Hui He et al. confirmed that lncRNA ZFPM2-AS1 acts as a miRNA sponge and promotes cell invasion through regulation of miR-139/growth differentiation factor 10 (GDF10) in HCC [7]. Our research team also discovered and studied the role of several lncRNAs in the progression of HCC in previous studies. For instance, we found that lncRNA CASC2 suppresses epithelial-mesenchymal transition of hepatocellular carcinoma cells through the CASC2/miR-367/ F-box and WD repeat domain containing 7 (FBXW7) axis [8]. Moreover, our study confirmed that MCM3AP-AS1 promotes hepatocellular carcinoma growth by targeting the miR-194-5p/ forkhead box A1 (FOXA1) axis [9]. Additional, the biological roles of lncRNA DSCR8 [10], AGAP2-AS1 [11], RUNX1-IT1 [12], and PICSAR [13] in HCC were investigated by our team. Here, we identified a novel upregulated lncRNA, MAPKAPK5-AS1, in HCC using The Cancer Genome Atlas (TCGA) data and Gene Expression Omnibus (GEO) datasets, and TCGA data also showed that MAPKAPK5-AS1 predicted poor prognosis of HCC patients. However, the expression, function, and exact mechanism of MAPKAPK5-AS1 is unknown.

Extensive work has reported that hypoxia, a hallmark of the solid tumor microenvironment, is involved in the progression of various cancers, including HCC [14–16]. Hypoxia-inducible factor 1α (HIF-1α), the key molecule in response to hypoxia, as a transcription factor, regulates the expression of a series of hypoxia-responsive genes, thereby mediating the effect of hypoxia on the biological behavior of cells [14, 17–20]. Recently, the effect of hypoxia on lncRNAs has attracted widespread attention, and increasing hypoxia-responsive lncRNAs are identified and studied. Libin Qiu et al. found that lncRNA DANCR alleviates hypoxia-caused H9c2 cells damage through the up-regulation of HIF-1α [21]. Lina Dong et al. confirmed that a positive feedback loop of lncRNA DSCR8/miR-98-5p/STAT3/HIF-1α plays a role in ovarian cancer [22]. Mingxing Hu et al. demonstrated that lncRNA HOTAIR knockdown inhibits glycolysis by regulating miR-130a-3p/ HIF-1α in HCC under hypoxia [23]. We also found that lncRNA RUNX1-IT1 downregulated by hypoxia-driven histone deacetylase 3 represses proliferation and cancer stem-like properties in HCC cells [12]. However, the effect of hypoxia on MAPKAPK5-AS1 expression is not explored.

In the present study, the expression level of MAPKAPK5-AS1 was detected. Subsequently, a series of in vitro and in vivo functional assays were carried out to investigate the biological roles of MAPKAPK5-AS1 in HCC. Next, the downstream mechanisms that mediate the functions of MAPKAPK5 were screened and verified. Finally, the effect of hypoxia on MAPKAPK5-AS1 expression was studied.

## Methods

### Tissue samples

Tissue samples used in this study were collected from 97 HCC patients at the First Affiliated Hospital of Xi’an Jiaotong University (Xi’an, China) between September 2014 to August 2015. Tumor and adjacent non-tumor tissue samples were immediately frozen in liquid nitrogen after being obtained and subsequently maintained in − 80 °C. The clinicopathological features of HCC patients were exhibited in Table 1.

**Table 1:** Association between MAPKAPK5-AS1 expression and clinicopathologic features of hepatocellular carcinoma patients

Characteristics	***n*** = 97	MAPKAPK5-AS1 levels		***p***-value
		High (***n*** = 49)	Low (***n*** = 48)	
Age (years)				0.774
≥ 60	64	33	31	
< 60	33	16	17	
Gender				0.255
Male	81	43	38	
Female	16	6	10	
HBV infection				0.184
Negative	19	7	12	
Positive	78	42	36	
Liver cirrhosis				0.240
Absent	29	12	17	
Present	68	37	31	
AFP (ng/ml)				0.305
≤ 20	22	9	13	
> 20	75	40	35	
Tumor size				0.006^*^
≤ 5 cm	41	14	27	
> 5 cm	56	35	21	
Tumor multiplicity				0.001^*^
Single	72	29	43	
Multiple	25	20	5	
Vascular invasion				0.008^*^
No	60	24	36	
Yes	37	25	12	
TNM stage				0.001^*^
I + II	78	33	45	
III + IV	19	16	3	

* indicates statistically significant

### Cell lines and cell culture

The human immortalized normal hepatic cell line (L02), HCC cell lines (Huh7, Hep3B, MHCC97-H, MHCC97-L, HepG2, HCCLM3), and HEK293T cells were obtained from the Cell Bank, Type Culture Collection, Chinese Academy of Sciences (Shanghai, China). All cells were cultured in Dulbecco’s modified Eagle medium (DMEM) (Gibco, Grand Island, NY, USA) containing 10% fetal bovine serum (FBS) (Gibco, Grand Island, NY, USA) and 1% penicillin-streptomycin (Sigma, St-Louis, MO, USA). All cell lines were cultured in a humidified incubator containing 5% CO_2_ at 37 °C. Hypoxia incubator (1% O2) was used to establish the hypoxic cell model.

### Cell transfection

sh-MAPKAPK5-AS1 (shRNA#1,2,3) and sh-negative control (sh-control) lentiviruses were purchased from GeneCreate Biological Engineering Co., Ltd. (Wuhan, China). The expression vector pcDNA3.1(+) (MAPKAPK5-AS1) and the empty plasmid pcDNA3.1(+) (Vector) were obtained from Invitrogen Corporation (Carlsbad, CA, USA). Hsa-miR-154-5p mimic (miR10000452–1-5) and hsa-miR-154-5p inhibitor (miR20000452–1-5) were purchased from RiboBio (Guangzhou, China). PLAGL2 Human cDNA ORF Clone (PLAGL2) and PLAGL2 siRNA (si-PLAGL2) were purchased from Technologies, Inc. (USA). The HIF-1α siRNA was purchased from Shanghai GenePharma Co., Ltd. (Shanghai, China). Cell transfection was performed according to the manufactures’ instructions.

### Quantitative real-time polymerase chain reaction (qRT-PCR)

TRIzol (Invitrogen) was used to extract total RNA from HCC tissues and cells following the manufacturer’s procedure. All miRNAs were reverse transcribed into cDNA with the miDETECT A Track miRNA qRT-PCR Starter Kit (C10712–1, RiboBio, Guangzhou, China). All lncRNAs and mRNAs were reverse transcribed into cDNA with the RevertAid First Strand cDNA Synthesis Kit (Thermo-Fisher Scientific). All primers used in this study were customized from RiboBio, and the sequence of primers are listed in Additional File 1: Table S1.

### MTT assay

Cells were added in a 96-well plate at a density of 1 × 10^4^/ml. 20 μl MTT solution (Sigma, St. Louis, MO, USA) was added to each well after incubation for 24, 48, 72, and 96 h. Then, the cells continued to be cultured for 4 h in a humidified incubator. After the supernatants were removed, 200 μl DMSO was added to each well. Finally, the absorbance was detected with a microplate reader (Bio-Rad, Hercules, CA, USA).

### Colony formation assay

Cells transfected for 48 h were plated in 6-well plates (1 × 10^3^/well). After cultured for 14 days, cell colonies were first fixed with 4% paraformaldehyde for 20 min, then stained with 0.5% crystal violet for 20 min, and finally photographed.

### Ethynyl deoxyuridine incorporation assay

Cell-Light EdU Apollo567 kit (Guangzhou RiboBio Co., Ltd.) was used to assess cell proliferation, and Zeiss fluorescence photomicroscope (Carl Zeiss, Oberkochen, Germany) was applied to acquire the results. EdU positive rate is calculated by counting at least five random fields.

### Cell apoptosis analysis

FITC Annexin V Apoptosis Detection Kit I was used to measure cell apoptosis in the study. And the assay was performed according to the manufactures’ instructions.

### Transwell assay

Transwell migration and invasion assays were conducted to evaluated cell mobility. These assays were conducted according to the protocols described in our previous studies [24].

### Western blotting

Protein extraction and western blotting assay were conducted according to the protocols described in our previous studies [24]. The primary antibodies used in this study are listed in Additional File 2: Table S2.

### Animal models

Subcutaneous xenograft model and lung metastasis model were established to assess the growth and metastasis ability of cells in vivo. Male BALB/C nude mice (4-week-old) were purchased from Shanghai SLAC Laboratory Animal Center of Chinese Academy of Sciences (Shanghai, China) and randomly divided into four groups (*n* = 6 per group). HCCLM3 cells transfected with sh-MAPKAPK5-AS1 and sh-control and Hep3B transfected with MAPKAPK5-AS1 and Vector were applied in our animal experiments. For subcutaneous xenograft model, transfected HCCLM3 and Hep3B cells were subcutaneously injected into the left flank of the mice, respectively (1 × 10^6^ per injection) (six mice per group). Tumor size was measured every 7 days, and tumor volume was calculated by the formula: tumor volume = 0.5 × (long diameter) × (short diameter)^2^. Tumors were removed for measurement after 4 weeks. For lung metastasis model, transfected HCCLM3 and Hep3B cells were injected via the tail vein (1 × 10^6^ per injection) (five mice per group). After 6 weeks, mice were sacrificed and the lung metastases were confirmed by hematoxylin and eosin (H&E) staining. The animal experiments were approved by the Animal Care and Use Committee of Xi’an Jiaotong University.

### Immunohistochemistry (IHC)

After the paraffin-embedded tumor tissue was sectioned, IHC staining for Ki67, E-Cadherin, N-Cadherin, and Vimentin was performed to assess the levels of the proteins according to the experimental instructions. Finally, the slides were scanned with the Leica slide scanning microscope imaging system. The result of Ki67 was assessed by the percentage of positive staining cells. The staining of epithelial-mesenchymal transition (EMT) markers were evaluated by IHC scores. The scoring is based on staining intensity: no staining scored 0, weakly staining scored 1, moderately staining scored 2, and strongly staining scored 3. The final score was calculated using the following percentage score × staining intensity score.

### Subcellular fractionation

The nuclear and cytoplasmic separation was performed with the PARIS Kit (Life Technologies, USA) according to the manufacturer’s instructions.

### Luciferase reporter assays

The wild-type (wt) or mutant (mut) sequence of MAPKAPK5-AS1 or 3’UTR of PLAGL2 was cloned into pGL3 vector (Promega, Madison, WI, USA), separately. The wt or mut luciferase plasmids and miRNA were co-transfected into HEK293T cells. The MAPKAPK5-AS1 promoters containing different HREs are cloned into pGL3-basic vector, and the pGL3-MAPKAPK5-AS1vectors were co-transfected with si-control or si-HIF-1α under hypoxia. According to the manufacturer’s instructions, the luciferase activity was detected with a Dual-Luciferase Reporter Assay System (Promega, USA) 48 h after transfection.

### Ago2-RNA immunoprecipitation (RIP) assay

The EZ-Magna RIP Kit (Millipore, Bedford, MA, USA) was used to performed RIP assay to explore the intracellular state of MAPKAPK5-AS1 and miR-154-5p. Anti-Ago2 and control IgG (Millipore, USA) antibodies were used in the assay. The coprecipitated MAPKAPK5-AS1 or miR-154-5p was evaluated by qRT-PCR.

### Chromatin Immunoprecipitation (ChIP)

ChIP assays were performed using a ChIP kit (CST, USA) to investigate the intracellular combination between HIF1 and HREs of MAPKAPK5-AS1 promoter. Cells were crosslinked with formaldehyde and sonicated to an average length of 200–1000 bp. Then, Immunoprecipitation was conducted with an anti-HIF-1α antibody (Abcam) or IgG control. Precipitated DNA was amplified by qRT-PCR using primers listed in Additional File 1: Table S1.

### Statistical analysis

Data of the study were shown as means ± SD, and at least three independent replicates were conducted. The differences were analyzed by Student’s *t*-test and analysis of variance using GraphPad Prism 7.0 (San Diego, CA, USA) and SPSS software 24.0 (SPSS Inc., Chicago, IL, USA). Statistical significance was evaluated via the Kaplan-Meier method, the log-rank test, and Pearson’s correlation analysis. *p* < 0.05 was deemed to indicate statistical significance.

## Results

### The differential expression and clinical significance of MAPKAPK5-AS1 in HCC

**Fig. 1:**
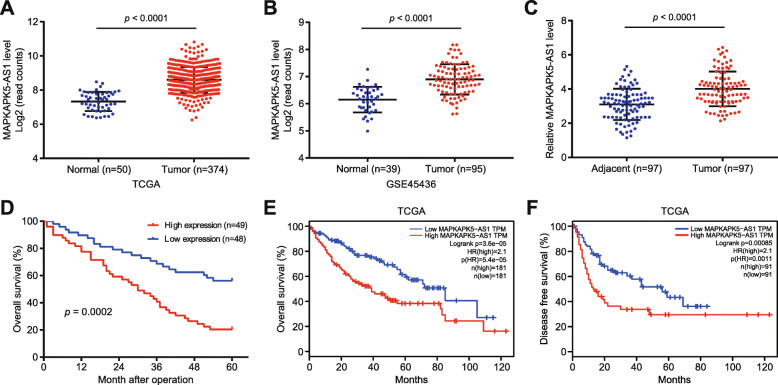
MAPKAPK5-AS1 upregulation is associated with poor clinical features and prognosis in HCC. **a** TCGA dataset showed the high expression level of MAPKAPK5-AS1 in HCC tissues compared to normal tissues. **b** GSE45436 dataset further exhibited high expression level of MAPKAPK5-AS1 in HCC tissues. **c** The level of MAPKAPK5-AS1 in 97 pairs HCC tissues and adjacent non-tumor tissues. **d** Kaplan-Meier survival analysis showed that HCC patients with high MAPKAPK5-AS1 expression exhibited a worse overall survival. **e**-**f** TCGA data showed that high MAPKAPK5-AS1 expression indicated poor overall survival (OS) and disease-free survival (DFS)

GEO and TCGA public databases were analyzed to identify lncRNAs which potentially exert vital biological function in HCC progression, and lncRNA MAPKAPK5-AS1 which was consistently elevated in HCC in three public databases, TCGA database (*p* < 0.0001, Fig. 1a), GSE45436 (*p* < 0.0001, Fig. 1b), and GSE54236 (*p* < 0.0001, Additional File 3: *Fig. S1A*), interested us. Subsequently, our qRT-PCR results confirmed the high expression of MAPKAPK5-AS1 in HCC tissues compared to adjacent non-tumor tissues (*p* < 0.0001, Fig. 1c). Furthermore, compared with human normal liver cells (L02), the MAPKAPK5-AS1 expression level was significantly upregulated in HCC cell lines (all *p* < 0.05, Additional File 3: Fig. S1B). Next, based on median MAPKAPK5-AS1expression in HCC tissues, patients were divided into MAPKAPK5-AS1 high or low-group to explore the clinical significance of MAPKAPK5-AS1 in HCC. Statistical analysis found that high MAPKAPK5-AS1 expression was significantly associated with large tumor size (*p* = 0.006), tumor multiplicity (*p* = 0.001), vascular invasion (*p* = 0.008) and advanced tumor-node-metastasis (TNM) stages (*p* = 0.001) (Table 1). Besides, TCGA data from UALCAN (http://ualcan.path.uab.edu/index.html) revealed that high MAPKAPK5-AS1 level had a positive association with the poor pathological stage and tumor grade of HCC patients (Additional File 3: Fig. S1C and S1D). Kaplan-Meier survival analysis for the 97 HCC patients showed that HCC patients with high MAPKAPK5-AS1 expression exhibited a notably worse overall survival (*p* = 0.0002, Fig. 1d). Moreover, TCGA data from GEPIA also showed that high MAPKAPK5-AS1 expression indicated poor overall survival (*p* = 3.6 × 10^− 5^, Fig. 1e) and disease-free survival (*p* = 8.5 × 10^− 4^, Fig. 1f). Altogether, these results demonstrated MAPKAPK5-AS1 was upregulated in HCC, which was associated with poor clinical features and prognosis of HCC patients.

### MAPKAPK5-AS1 contributes to the proliferation and suppresses apoptosis of HCC cells in vitro

**Fig. 2:**
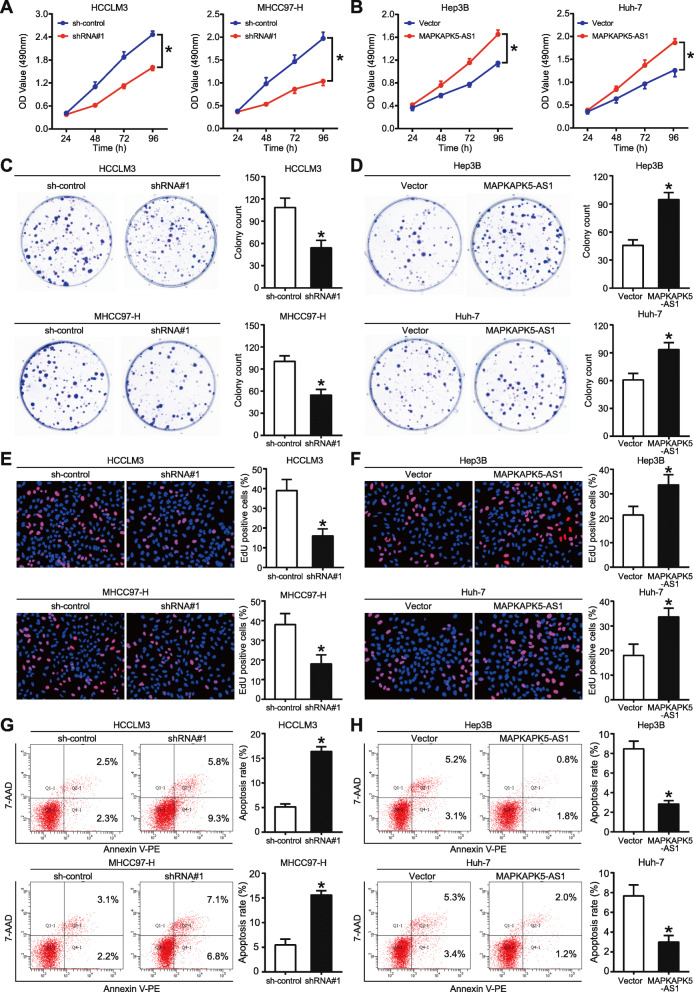
MAPKAPK5-AS1 promotes proliferation and inhibits apoptosis of HCC cells. **a**-**b** MTT assay was carried out to examined the effect of MAPKAPK5-AS1 knockdown or overexpression on HCC cells viability. **c**-**d** Colony formation confirmed the negative regulatory effect of MAPKAPK5-AS1 on tumor cells proliferation. **e**-**f** The influence of MAPKAPK5-AS1 on DNA synthesis activity of HCC cell was evaluated by EdU assay. **g**-**h** The effect of MAPKAPK5-AS1 on cell apoptosis was assessed by flow cytometry. *p < 0.05

To study the biological functions of MAPKAPK5-AS1, we stably knocked down MAPKAPK5-AS1 in HCCLM3 and MHCC97-H cells and overexpressed MAPKAPK5-AS1 in Hep3B and Huh-7 cells. qRT-PCR results indicating the transfection efficiency of these four HCC cell lines with MAPKAPK5-AS1 overexpression or knockdown are exhibited in Additional File 4: Fig. S2. In MTT, colony formation, and EdU assays, MAPKAPK5-AS1 knockdown significantly inhibited viability and proliferation of HCCLM3 and MHCC97-H cells (*p* < 0.05, Fig. 2a, c, and e), whereas the viability and proliferation of Hep3B and Huh-7 cells were markedly enhanced by MAPKAPK5-AS1 overexpression (*p* < 0.05, Fig. 2b, d, and f). Besides, flow cytometry analysis demonstrated that MAPKAPK5-AS1 deficiency in HCCLM3 and MHCC97-H cells obviously induced cell apoptosis (*p* < 0.05, Fig. 2g), while cell apoptosis was strongly suppressed by MAPKAPK5-AS1 overexpression in Hep3B and Huh-7 cells (*p* < 0.05, Fig. 2h). These results above indicated that MAPKAPK5-AS1 promoted the proliferation of HCC cells by suppressing cell apoptosis.

### MAPKAPK5-AS1 facilitates the mobility and EMT process of HCC cells in vitro

**Fig. 3:**
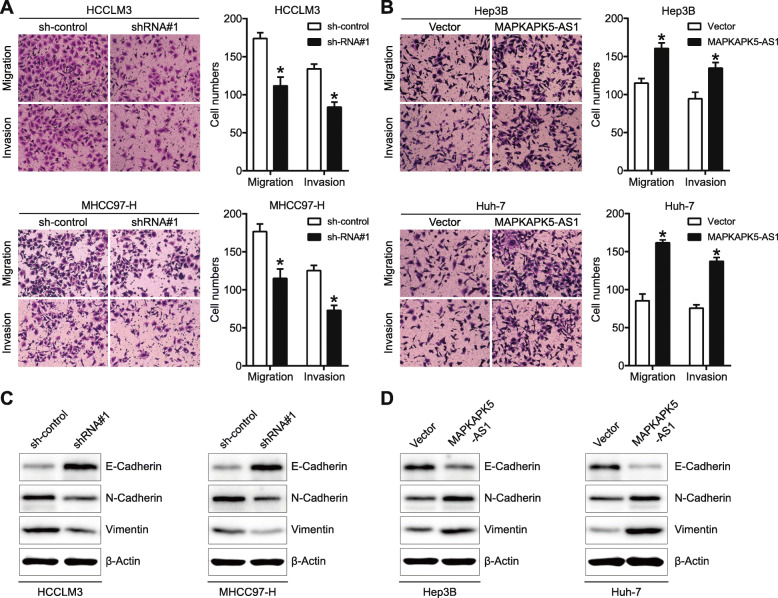
MAPKAPK5-AS1 enhances migration, invasion, and EMT process of HCC cells. **a**-**b** Transwell assay were performed to evaluate the effects of MAPKAPK5-AS1 on the tumor cells abilities of migration and invasion. **c**-**d** Expression of EMT markers was detected by western blotting to assess the effect of MAPKAPK5-AS1 on EMT process. *p < 0.05

Next, transwell migration and invasion assays were carried out to explore the effect of MAPKAPK5-AS1 on cells mobility, and the results showed that MAPKAPK5-AS1 knockdown in HCCLM3 and MHCC97-H remarkably curbed the migration and invasion of tumor cells (*p* < 0.05, Fig. 3a), whereas the mobility of Hep3B and Huh-7 cells was notably enhanced by overexpressing MAPKAPK5-AS1 (*p* < 0.05, Fig. 3b). Considering that EMT is a curial mechanism mediating cancer metastasis, the effect of MAPKAPK5-AS1 on the EMT process of tumor cells was further investigated. Western blotting assays showed that MAPKAPK5-AS1 knockdown in HCCLM3 and MHCC97-H substantially increased the expression of epithelial markers (E-cadherin) and decreased the level of the mesenchymal markers (N-cadherin and Vimentin) (Fig. 3c). The opposite regulatory effect on EMT markers expression was observed after overexpressing MAPKAPK5-AS1 in Hep3B and Huh-7 cells (Fig. 3d). Thus, our above results demonstrated that MAPKAPK5-AS1 contributed to HCC cells’ mobility by regulating the EMT process.

### MAPKAPK5-AS1 promotes the growth and metastasis of HCC cells in vivo

**Fig. 4:**
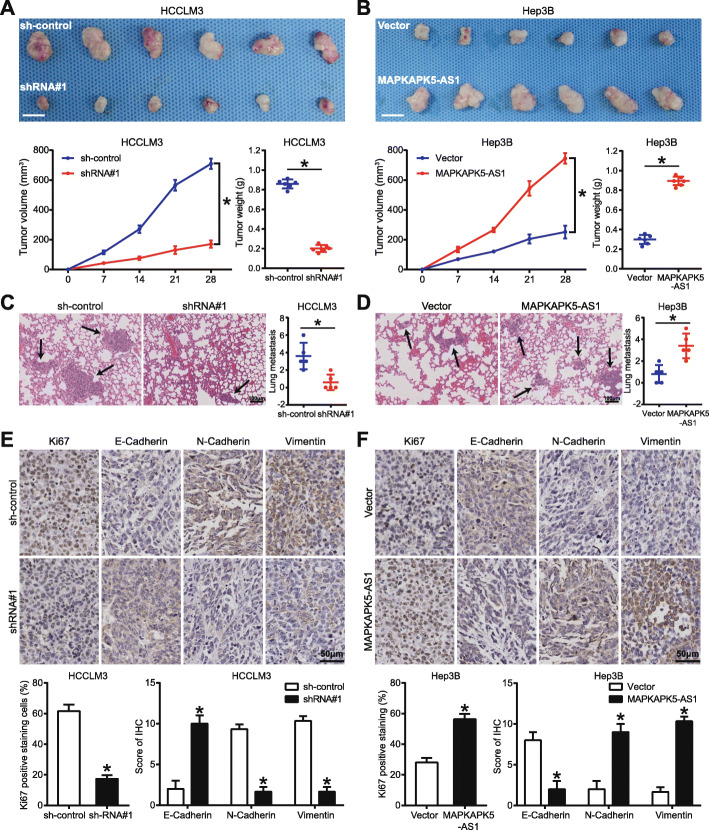
MAPKAPK5-AS1 promotes the growth and metastasis of HCC cell in vivo*.* **a**-**b** Xenograft tumor model showed the negative regulatory effect of MAPKAPK5-AS1 on tumor size, growth rate, and tumor weight. **c**-**d** Representative images of tail vein injection model and the number of lung metastatic nodes. **e**-**f** IHC results of Ki67 and EMT markers for xenograft tumors. *p < 0.05

These above mentioned in vitro experimental results indicate that MAPKAPK5-AS1 functions as an oncogene in HCC progression. To make the conclusion more credible, we performed various in vivo experiments to investigate the effects of MAPKAPK5-AS1 on the growth and metastasis of HCC cells. HCCLM3 cells with MAPKAPK5-AS1 knockdown or Hep3B cells with MAPKAPK5-AS1 overexpression were implanted into nude mice via subcutaneous injection or tail vein injection. The results indicated that knocking down MAPKAPK5-AS1 in HCCLM3 cells remarkably suppressed tumor cells’ growth and lung metastasis in vivo compared with the control group (*p* < 0.05, Fig. 4a and c). By contrast, overexpressing MAPKAPK5-AS1 in Hep3B cells significantly promoted tumor cells’ growth and lung metastasis in vivo (*p* < 0.05, Fig. 4b and d). Subsequently, RNA in subcutaneous tumors was extracted to detect the expression of MAPKAPK5-AS1 by RT-qPCR assay, and the results showed that the level of MAPKAPK5-AS1 in tumor tissues arising from the MAPKAPK5-AS1 knockdown group was significantly lower than that in the control group (*p* < 0.05, Additional File 5: Fig. S3A), and the level of MAPKAPK5-AS1 in tumor tissues arising from MAPKAPK5-AS1 overexpression group was significantly higher than that in the control group (*p* < 0.05, Additional File 5: Fig. S3B). To further validate the influence of MAPKAPK5-AS1 on HCC cell growth and EMT phenotype, we performed IHC analysis for tumors formed by different HCC cells. Results showed that knocking down MAPKAPK5-AS1 significantly decreased the positive rate of Ki67 staining, increased the expression of E-cadherin, and decreased the level of N-cadherin and vimentin in tumors formed by HCCLM3 cells (*p* < 0.05, Fig. 4e). By contrast, overexpressing MAPKAPK5-AS1 in Hep3B cells promoted the growth and EMT progression (*p* < 0.05, Fig. 4f). Together, these results confirmed that MAPKAPK5-AS1 promoted HCC cell growth and metastasis in vivo.

### MAPKAPK5-AS1 sponges miR-154-5p in HCC cells

**Fig. 5:**
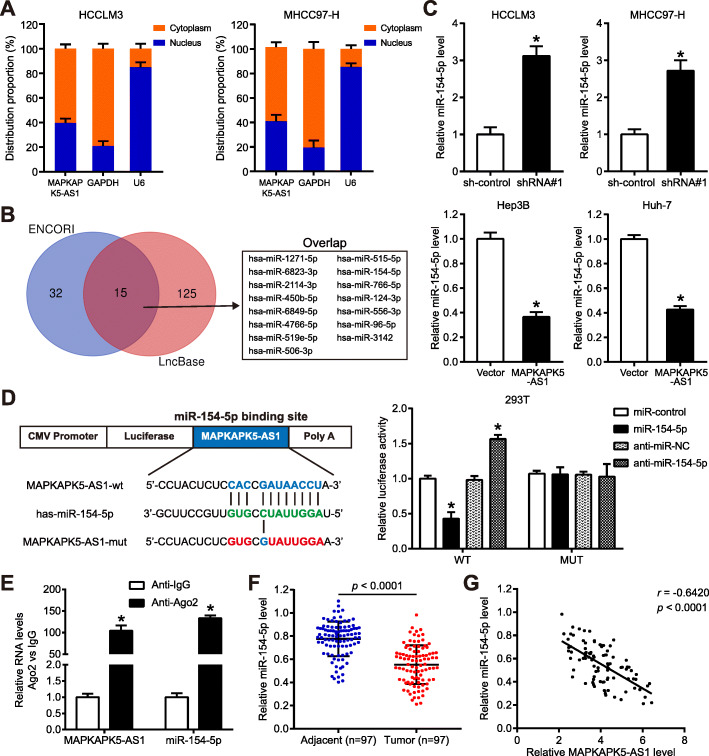
MAPKAPK5-AS1 acts as a sponge of miR-154-5p in HCC cells. **a** Subcellular fractionation assay was applied to determine MAPKAPK5-AS1 subcellular localization. **b** 15 putative targets of MAPKAPK5-AS1 was predicted by applying two bioinformatics prediction tools. **c** qRT-PCR showed that MAPKAPK5-AS1 negatively regulated miR-154-5p expression in HCC cells. **d** The predicted binding sites between miR-154-5p and MAPKAPK5-AS1 and mutant sequences of the potential miR-154-5p binding site in MAPKAPK5-AS1. Additionally, the result of luciferase reporter assay is shown here. (E) Anti-Ago2 RIP assay was performed in HCCLM3 with miR-154-5p overexpression, followed by qRT-PCR to detect the level of MAPKAPK5-AS1 or miR-154-5p associated with Ago2. **f** The expression level of miR-154-5p in HCC tissues and adjacent non-tumor tissues. **g** The relativity between MAPKAPK5-AS1 and miR-154-5p in HCC tissues. *p < 0.05

LncRNAs regulate gene expression at the transcriptional level or post-transcriptional level, depending on lncRNAs subcellular localization. A subcellular fractionation assay was applied to investigate the molecular mechanism involved in the MAPKAPK5-AS1 function. Results indicated that MAPKAPK5-AS1 was mainly located in the cytoplasm of HCC cells (Fig. 5a and Additional File 6: Fig. S4A), which suggested that MAPKAPK5-AS1 may exert its biological function through its mechanism of competitive endogenous RNA (ceRNA). Then, ENCORI and LncBase, these two online bioinformatics websites, were used to predict potential targeted miRNAs for MAPKAPK5-AS1. Fifteen miRNAs were overlapping between these two predicted lists among these predicted miRNAs (Fig. 5b). Previous studies showed that only miR-1271–5p [25, 26], miR-154-5p [27–29], and miR-450b-5p [30, 31], these three miRNAs, were down-regulated and acted as tumor suppressor genes. To determine which miRNA can actually be regulated by MAPKAPK5-AS1, we detected these three miRNAs expression levels respectively after knocking down MAPKAPK5-AS1 in HCC cells. Our data revealed that only miR-154-5p could be significantly negatively regulated by MAPKAPK5-AS1 in HCC cells (*p* < 0.05, Additional File 6: Fig. S4B; *p* < 0.05, Fig. 5c). Next, Ago2-RIP and luciferase reporter assays were performed to identify whether there was direct interaction between MAPKAPK5-AS1 and miR-154-5p. Firstly, the Ago2-RIP assay showed that the Ago2 antibody could pull down both endogenous MAPKAPK5-AS1 and miR-154-5p, which confirmed that MAPKAPK5-AS1 and miR-154-5p could bind with Ago2 in RISC (*p* < 0.05, Fig. 5e). Secondly, luciferase reporter assay showed that miR-154-5p could significantly negatively regulate the luciferase activity of vectors containing wild-type MAPKAPK5-AS1 but failed to reduce that of the mutant vector (*p* < 0.05, Fig. 5d), which demonstrated that miR-154-5p was directly bound to MAPKAPK5-AS1. Subsequently, the level of miR-154-5p in paired tissue samples was detected. As expected, miR-154-5p was down-regulated in HCC tissue (*p* < 0.0001, Fig. 5f and Additional File 6: Fig. S4C) and exhibited a negative correlation with the expression of MAPKAPK5-AS1 (*r* = − 0.6420, *p* < 0.0001, Fig. 5g). These results illustrated that MAPKAPK5-AS1 acted as a molecular sponge for miR-154-5p in HCC cells.

### MiR-154-5p mediates the biological function of MAPKAPK5-AS1

**Fig. 6:**
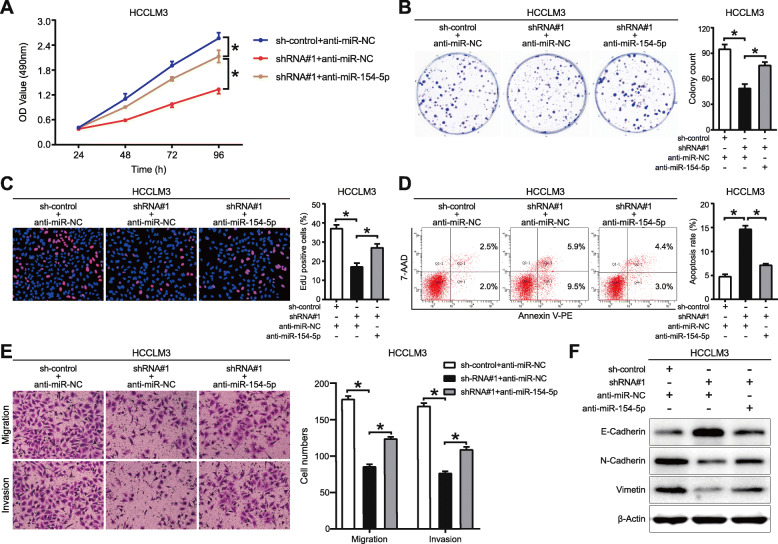
MiR-154-5p mediates the function of MAPKAPK5-AS1. **a**-**c** MTT, colony formation and EdU assays were conducted to explore the effect of miR-154-5p inhibitors on cell proliferation suppressed by MAPKAPK5-AS1 knockdown. **d** Flow cytometry showed the effect of miR-154-5p inhibitors on apoptosis induced by MAPKAPK5-AS1 knockdown. **e**-**f** Transwell assay were performed to evaluate the effects of miR-154-5p inhibitors on the tumor cells abilities of migration and invasion impaired by MAPKAPK5-AS1 kmockdown. **c**-**d** Expression of EMT markers was detected by western blotting to assess the effect of miR-154-5p inhibitors on EMT process inhibited by MAPKAPK5-AS1 knockdown. *p < 0.05

Recuse assays were conducted to explore whether miR-154-5p mediated the biological function of MAPKAPK5-AS1. The results of MTT, colony formation, and EdU assays revealed that miR-154-5p inhibitor partially reversed the anti-proliferation effect of sh-MAPKAPK5-AS1 (*p* < 0.05, Fig. 6a-c), while miR-154-5p mimics could impair the pro-proliferation effect of MAPKAPK5-AS1(*p* < 0.05, Additional File 7: Fig. S5A-C). Additionally, flow cytometry analysis results showed that cell apoptosis induced by MAPKAPK5-AS1 knockdown was inhibited by co-transfecting with miR-154-5p inhibitor (*p* < 0.05, Fig. 6d) while overexpressing miR-154-5p induced cell apoptosis inhibited by MAPKAPK5-AS1 (*p* < 0.05, Additional File 7: Fig. S5D). Subsequently, transwell migration and invasion assays exhibited that the motility decreased by knocking down MAPKAPK5-AS1 was driven by inhibiting miR-154-5p expression in HCCLM3 cells (*p* < 0.05, Fig. 6e), whereas the motility enhanced by MAPKAPK5-AS1 overexpression was impaired by miR-154-5p mimics in Hep3B cells (*p* < 0.05, Additional File 7: Fig. S5E). Similarly, western blot assay confirmed that the EMT progression hindered by MAPKAPK5-AS1 knockdown was partially recovered by a miR-154-5p inhibitor (*p* < 0.05, Fig. 6f), whereas the EMT progression promoted by MAPKAPK5-AS1 was partially abolished by miR-154-5p mimics (*p* < 0.05, Additional File 7: Fig. S5F). These recuse assays confirmed that MAPKAPK5-AS1 exerted its biological function through sponging miR-154-5p in HCC cells.

### PLAGL2 is the direct target of miR-154-5p

**Fig. 7:**
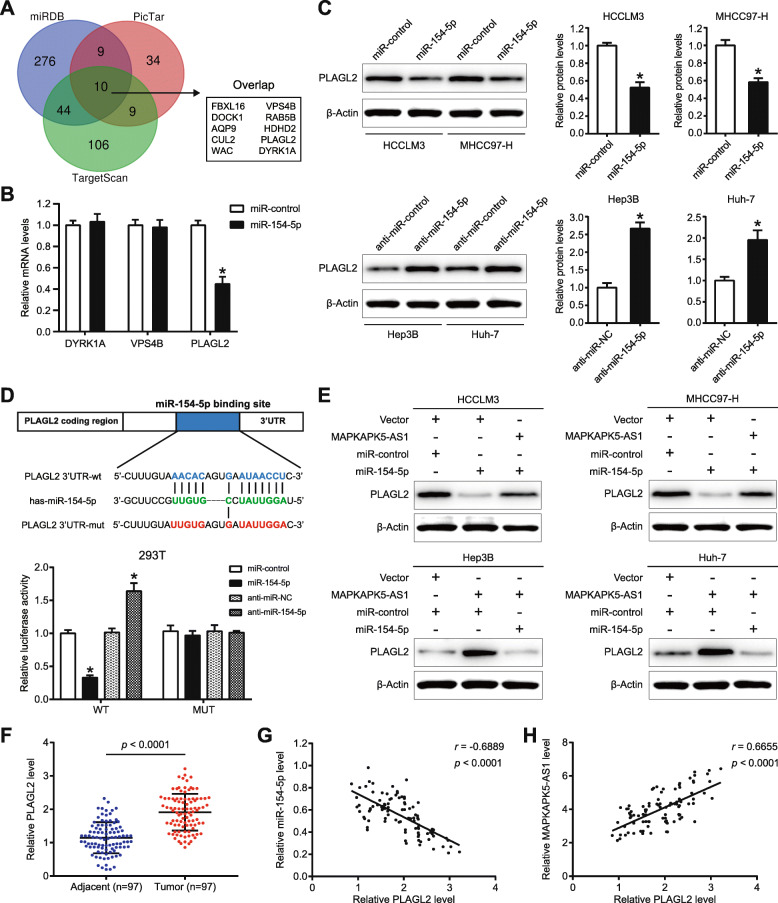
PLAGL2 is the direct functional target of miR-154-5p. **a** 10 potential targets of miR-154-5p was predicted by applying three online bioinformatics tools. **b** mRNA levels of three potential targets in Hep3B cell with miR-154-5p overexpression. **c** The effect of miR-154-5p on PLAGL2 protein level evaluated by western blotting. **d** The predicted binding sites between miR-154-5p and PLAGL2 and mutant sequences of the potential miR-154-5p binding site in PLAGL2. In addition, the result of luciferase reporter assay is exhibited. **e** Western blotting confirmed that MAPKAPK5-AS1 and miR-154-5p could reverse the regulatory effect on PLAGL2 expression reciprocally. **f** The expression level of PLAGL2 in HCC tissues and adjacent non-tumor tissues. **g** Expression association between PLAGL2 and miR-154-5p in HCC tissues. **h** Expression association between PLAGL2 and MAPKAPK5-AS1 in HCC tissues. *p < 0.05

Considering that miRNAs exert its function by modulating the expression of target genes, miRDB, PicTar, and TargetScan, these three bioinformatics databases, were used to predict the potential targets of miR-154-5p. As shown in Fig. 7a, ten targets were overlapping in the prediction results of these three databases. Among these genes, only DYRK1A [32], VPS4B [33], PLAGL2 [34], these three genes have been reported to act as tumor-promoter in HCC. Then, we detected the three mRNAs’ levels in HCCLM3 cells transfected with miR-154-5p mimics, and we found that just PLAGL2 mRNA could be significantly negatively altered (*p* < 0.05 Fig. 7b). Subsequently, we found that miR-154-5p mimics significantly decreased PLAGL2 mRNA and protein levels in both HCCLM3 and MHCC97-H cells (*p* < 0.05, Additional File 8: Fig. S6A and Fig. 7c). Also, PLAGL2 mRNA and protein expression were elevated by miR-154-5p inhibitor in both Hep3B and HuH-7 cells (*p* < 0.05, Additional File 8: Fig. S6B and Fig. 7c). These data confirmed that PLAGL2 expression could be negatively regulated by miR-154-5p in HCC cells. Next, we attempted to explore whether there exists direct binding between miR-154-5p and PLAGL2 mRNA. The putative binding sequence was listed in Fig. 7d. Further luciferase reporter assay showed that miR-154-5p mimics or inhibitors could remarkably negatively modulate the luciferase activity of wild-type PLAGL2 vector but not the mutant vector (*p* < 0.05, Fig. 7d). Furthermore, we found that MAPKAPK5-AS1 could partially abolish the inhibitory effect of miR-154-5p on PLAGL2 protein expression in both HCCLM3 and MHCC97-H cells, and miR-154-5p also could partially reverse the promotional effect of MAPKAPK5-AS1 on PLAGL2 protein expression in both Hep3B and HuH-7 cells (Fig. 7e). Moreover, we observed that PLAGL2 level was elevated in HCC tissues compared with adjacent non-tumor tissues (*p* < 0.0001, Fig. 7f and Additional File 8: *Fig. S*6C). Additionally, statistical analysis showed that the expression of PLAGL2 was negatively correlated with miR-154-5p expression (*r* = − 0.6889, *p* < 0.0001, Fig. 7g), while positively correlated with MAPKAPK5-AS1 expression (*r* = 0.6655, *p* < 0.0001, Fig. 7h; *r* = 0.294, *p* < 0.0001, Additional File 8: Fig. S6D). Finally, the PLAGL2 expression in xenograft tumors was detected by western blotting, and the results showed that MAPKAPK5-AS1 could positively modulate the expression of PLAGL2 in xenograft tumors (Additional File 8: Fig. S6E). Therefore, our data indicated that MAPKAPK5-AS1 positively altered PLAGL2 expression by inhibition of miR-154-5p.

### PLAGL2/EGFR/AKT pathway mediates the tumor-promoting effect of MAPKAPK5-AS1

**Fig. 8:**
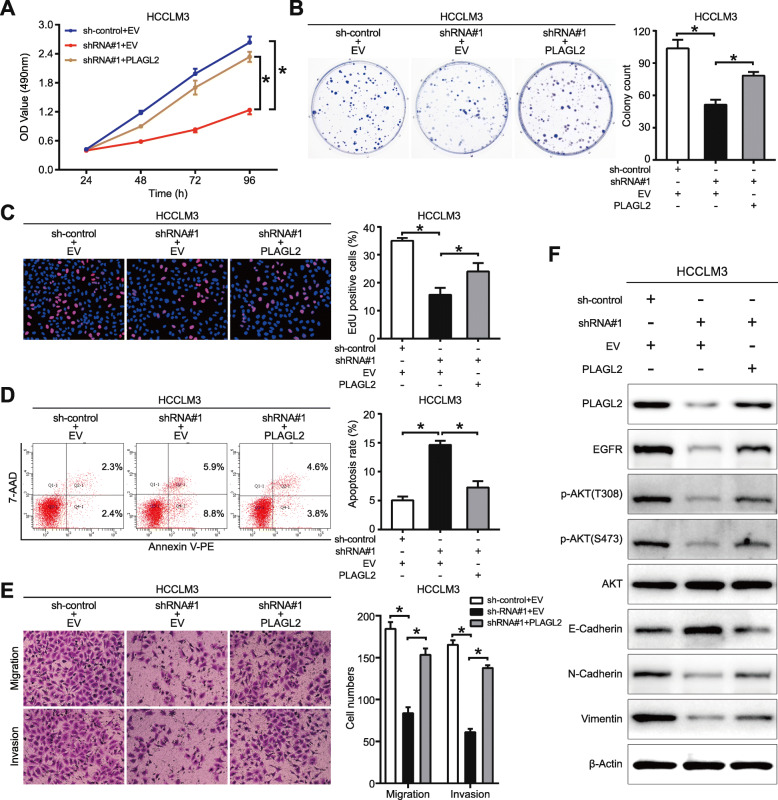
PLAGL2/EGFR/AKT pathway mediates the oncogenic effect of MAPKAPK5-AS1. **a**-**c** MTT, colony formation and EdU assays showed the effect of PLAGL2 on cell proliferation suppressed by MAPKAPK5-AS1 knockdown. **d** Flow cytometry showed the effect of PLAGL2 on cell apoptosis induced by MAPKAPK5-AS1 knockdown. **e** Transwell assay showed the effects of PLAGL2 on migration and invasion of the tumor cells suppressed by MAPKAPK5-AS1 knockdown. **f** Expression of EMT and EGFR/AKT pathway markers was detected by western blotting to assess the effect of PLAGL2 on EMT process and pathway activity inhibited by MAPKAPK5-AS1 knockdown. *p < 0.05

Given that previous research has confirmed that PLAGL2 functions as a transcriptional regulator of EGFR and promotes HCC cell proliferation, migration, and invasion through the EGFR-AKT pathway [34], we have determined that MAPKAPK5-AS1 upregulates PLAGL2 level by sponging miR-154-5p. Recuse assays were conducted to investigate whether PLAGL2/EGFR/AKT mediated the biological function of MAPKAPK5-AS1. The results of MTT, colony formation, and EdU assays revealed that restriction of MAPKAPK5-AS1 knockdown on cell proliferation was partially repealed by PLAGL2 overexpression in HCCLM3 (*p* < 0.05, Fig. 8a-c), and PLAGL2 knockdown reversed the promoting effect of MAPKAPK5-AS1 on proliferation (*p* < 0.05, Additional File 9: *Fig. S*7A-C). In addition, we found that MAPKAPK5-AS1 knockdown induced HCCLM3 cell apoptosis, which could be partially abolished by PLAGL2 overexpression (*p* < 0.05, Fig. 8d). Similarly, knocking down PLAGL2 in Hep3B cells attenuated the inhibitory effect of MAPKAPK5-AS1 on cell apoptosis (*p* < 0.05, Additional File 9: Fig. S7D). Subsequently, results of transwell and western blot assays indicated that the motility and EMT of HCCLM3 weakened by MAPKAPK5-AS1 knockdown was enhanced by PLAGL2 (*p* < 0.05, Fig. 8e and f), and the motility and EMT of Hep3B enhanced by MAPKAPK5-AS1 were weakened by PLAGL2 knockdown (*p* < 0.05, Additional File 9: Fig. S7E and 7F). Moreover, we also found that silencing MAPKAPK5-AS1 inactivated epidermal growth factor receptor (EGFR)/AKT signaling in HCCLM3 cells, which could be reversed by PLAGL2 (Fig. 8f), and vice versa in Hep3B cells (Additional File 9: Fig. S7F). In addition, a series of experiments were performed to detect the effect of MK2206, the inhibitor of AKT, on the tumor-promoting effect of MAPKAPK5-AS1, and the results showed that MAPKAPK5-AS1 overexpression enhanced the proliferation, the motility and EMT of Hep3B, and reduced Hep3B cell apoptosis, which could be partially reserved by MK2206 treatment (Additional File 10: *Fig. S8*). Overall, these recuse experiments demonstrated that PLAGL2/EGFR/AKT pathway mediated the biological function of MAP-KAPK5-AS1 in HCC cells.

### Hypoxia regulates a MAPKAPK5-AS1/PLAGL2/HIF-1α signaling loop

**Fig. 9:**
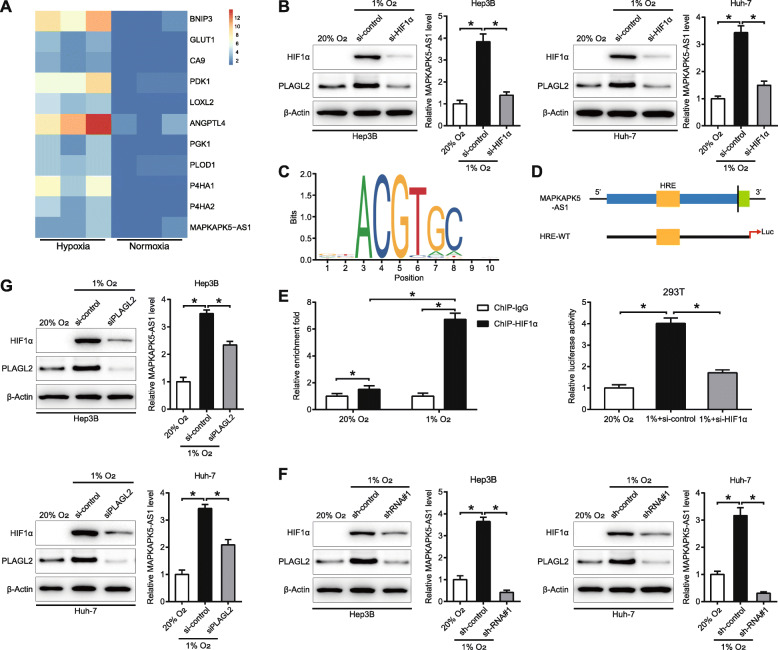
Hypoxia triggers a MAPKAPK5-AS1/PLAGL2/HIF-1α signaling loop. **a** Heat map of RNA-seq data exhibited the upregulation of MAPKAPK5-AS1 under hypoxia. **b** The upregulation of MAPKAPK5-AS1 and PLAGL2 induced by hypoxia was suppressed by HIF-1α knockdown. **c** The recognition motif of HIF-1α from JSAPAR database. **d** The hypoxia responsive element (HRE) in MAPKAPK5-AS1 promoter. In addition, the result of luciferase reporter assay is exhibited. **e** ChIP assay was conducted to validate the binding between HIF-1α and the HRE of the lncRNA MAPKAPK5-AS1 promoter under normoxia and hypoxia. **f** The regulatory effect of MAPKAPK5-AS1 on HIF-1α and PLAGL2 level. **d** The effect of PLAGL2 on HIF-1α and MAPKAPK5-AS1 expression under hypoxia. *p < 0.05

Considering that PLAGL2 has been reported to be a hypoxia-responsive gene, we investigated whether hypoxia was involved in MAPKAPK5-AS1 expression regulation based on our above research results. Our lncRNA microarray data (GSE155505) showed that the MAPKAPK5-AS1 level was elevated under hypoxic conditions (Fig. 9a), and this finding was supported by qRT-PCR (*p* < 0.05, Fig. 9b). In addition, western blotting assays exhibited that hypoxia induced the upregulation of PLAGL2 (Fig. 9b), which was consistent with the previous study [34]. Furthermore, we found that HIF-1α knockdown significantly impaired the upregulation of MAPKAPK5-AS1 and PLAGL2 induced by hypoxia (*p* < 0.05, Fig. 9b), which indicated that HIF-1α mediated the regulation of hypoxia on the expression of MAPKAPK5-AS1 and PLAGL2. Next, JASPAR webtool predicted a potential hypoxia-response element (HRE) in the promoter of MAPKAPK5-AS1 (Fig. 9c). Consequently, we established a luciferase report vector containing the MAPKAPK5-AS1 promoter. And the results showed that hypoxia remarkably enhanced the luciferase activity in cells transfected with the vector containing MAPKAPK5-AS1 promoter, and HIF-1α knockdown could reverse the luciferase activity induced by hypoxia, which suggested MAPKAPK5-AS1 promoter containing the functional HRE (*p* < 0.05, Fig. 9d). Moreover, the ChIP assay further confirmed the direct binding between HIF-1α and HRE in the MAPKAPK5-AS1 promoter (*p* < 0.05, Fig. 9e). Then the regulation of MAPKAPK5-AS1 on HIF-1α and PLAGL2 was studied. Western blotting assays revealed that the upregulation of HIF-1α and PLAGL2 induced by hypoxia was obviously attenuated by MAPKAPK5-AS1 knockdown (*p* < 0.05, Fig. 9f). Finally, we explored the effect of PLAGL2 on HIF-1α and MAPKAPK5-AS1. The results showed that hypoxia elevated the level of HIF-1α and MAPKAPK5-AS1, which could be partly reversed by PLAGL2 knockdown (*p* < 0.05, Fig. 9g). Our results collectively indicated that HIF-1α could directly bind to the promoter to activate MAPKAPK5-AS1 transcription, and MAPKAPK5-AS1 regulated HIF-1α expression through PLAGL2 form a hypoxia-mediated MAPKAPK5-AS1/PLAGL2/HIF-1α signaling loop in HCC.

## Discussion

Recently, increasing lncRNAs involved in hepatocellular carcinoma progression have been reported [35–37], which provides us with a new perspective to understand tumor occurrence and development, thereby offering us new targets for treating hepatocellular carcinoma. LncRNA MAPKAPK5-AS1 is identified as a novel critical oncogene in colorectal cancer [38]. However, the role of MAPKAPK5-AS1 in HCC has not been investigated. Here, we showed that lncRNA MAPKAPK5-AS1 expression was elevated in HCC, and its high expression was significantly positively associated with poor clinical features and prognosis of hepatocellular carcinoma patients. Functionally, MAPKAPK5-AS1 acts as a tumor-promotor through contributing to HCC cell growth and metastasis in vitro and in vivo. Our study identified the oncogenic role of MAPKAPK5-AS1 for the first time in hepatocellular carcinoma.

The subcellular localization of lncRNAs can indicate the mechanism mediating their biological functions. Studies have shown that lncRNAs located in the cell nucleus mainly regulate gene expression through binding with various proteins. In contrast, lncRNAs, which are located in the cytoplasm, generally regulate gene expression at the post-transcriptional level through the ceRNA mechanism [39]. In the study, we observed that MAPKAPK5-AS1 was mainly located in the cytoplasm by subcellular fractionation assay, which suggested that MAPKAPK5-AS1 might exert its oncogenic role in HCC cells through functioning as a miRNA sponge. Therefore, we next predicted the potential target of MAPKAPK5-AS1, miR-154-5p, by online bioinformatics website, which was further verified through luciferase reporter assay and Ago2-RIP assay. In addition, we found that there was a negative correlation between miR-154-5p and MAPKAPK5-AS1 expression in HCC tissue. Significantly, we performed a series of recusing assays, which demonstrated that miR-154-5p mediated the oncogenic function of MAPKAPK5-AS1 in HCC cells. In summary, we determined that MAPKAPK5-AS1 could sponge miR-154-5p in HCC cells.

Previous studies have reported that pleomorphic adenoma gene like-2 (PLAGL2), as a member of the PLAG zinc finger transcription factor, is widely involved in the progress of various tumors [40–42], including HCC. For example, Liang Wu et al. confirmed that PLAGL2 promoted epithelial-mesenchymal transition and mediated colorectal cancer metastasis via β-catenin-dependent regulation of ZEB1 [42]. And Weiwei Hu et al. found that PLAGL2 regulated the transcription of EGFR, enhanced the PI3K/PDK1/mTORC2-dependent AKT phosphorylation, and upregulated expression of HIF-1/2α [34, 43]. In the present study, we discovered a new mechanism regulating PLAGL2 expression at the post-transcriptional level under hypoxia. Firstly, we identified PLAGL2 as the direct target of miR-154-5p in HCC cells through bioinformatic prediction and experimental verification. Secondly, we found that PLAGL2 was positively modulated by MAPKAPK5-AS1 in HCC cells. Thirdly, we found the expression of PLAGL2 was negatively correlated with miR-154-5p expression while positively correlated with MAPKAPK5-AS1 expression in HCC tissues. Finally, we conducted a series of recusing assays to illustrate that PLAGL2 mediated the oncogenic function of MAPKAPK5-AS1 in HCC cells. Taken together, we demonstrated that MAPKAPK5-AS1 could promote PLAGL2 expression by sponging miR-154-5p in HCC.

**Fig. 10:**
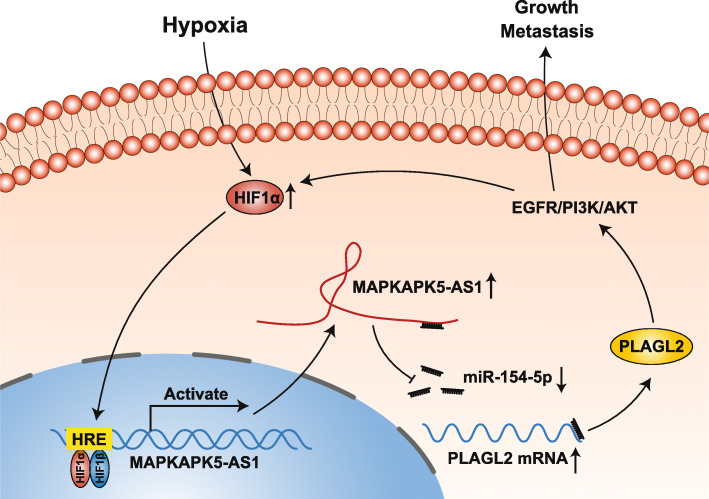
Schematic of the findings of the present study

Hypoxia, a significant feature of solid tumors, widely affects various tumors [44–46]. Studies have found that many hypoxia-responsive lncRNAs are involved in the impact of hypoxia on tumor biological behavior. For example, Aijun Yu et al. confirmed that the lncRNA H19/microRNA-612/Bcl-2 axis mediates the promotion of HIF-1α on proliferation, migration, and invasion in cholangiocarcinoma [47]. In the current study, we investigated the effect of hypoxia on MAPKAPK5-AS1 expression for the first time. Firstly, we observed that hypoxia could induce MAPKAPK5-AS1 expression, which could be significantly impaired by HIF-1α knockdown. Subsequently, we predicted a potential HRE in the promoter of MAPKAPK5-AS1 through the JASPAR database. Moreover, we confirmed the regulatory effect of HIF-1α on MAPKAPK5-AS1 transcription in response to hypoxia by ChIP and dual-luciferase reporter assays. Next, our results revealed that the upregulation of HIF-1α and PLAGL2 induced by hypoxia was obviously attenuated by MAPKAPK5-AS1 knockdown, and hypoxia elevated the level of HIF-1α and MAPKAPK5-AS1, which could be partly reversed by PLAGL2 knockdown. These results indicated that HIF-1α, as a transcript factor, could directly bind to the MAPKAPK5-AS1 promoter to activate its transcription, and MAPKAPK5-AS1 regulates HIF-1α expression through PLAGL2 to form a hypoxia-mediated MAPKAPK5-AS1/PLAGL2/HIF-1α signaling loop in HCC (Fig. 10).

## Conclusions

In the current study, we identified an upregulated lncRNA MAPKAPK5-AS1, associated with poor clinical features and prognosis of HCC. Moreover, MAPKAPK5-AS1 contributed to the growth and metastasis of HCC cells through the miR-154-5p/PLAGL2/EGRT/AKT axis. Furthermore, our results indicate that HIF-1α, as a transcript factor, could directly bind to the MAPKAPK5-AS1 promoter to activate its transcription, and MAPKAPK5-AS1 regulates HIF-1α expression through PLAGL2 to form a hypoxia-mediated MAPKAPK5-AS1/PLAGL2/HIF-1α signaling loop in HCC. In summary, the study reveals the role of MAPKAPK5-AS1 in HCC progression, and it could be a potential therapeutic target and prognostic predictor of HCC.

## Supplementary Information

**Additional file 1.**

**Additional file 2.**

**Additional file 3: Figure S1.** The expression and clinical significance of MAPKAPK5-AS1 in HCC. (A) GSE54236 dataset showed the high expression level of MAPKAPK5-AS1 in HCC tissues compared to normal tissues. (B) The level of MAPKAPK5-AS1 in human normal liver cell (L02) and HCC cell lines. (C) The association between MAPKAPK5-AS1 expression and pathological stage of HCC patients. (D) The association between MAPKAPK5-AS1 expression and tumor grade of HCC patients. *p < 0.05.

**Additional file 4: Figure S2.** Transfection efficiency of these four HCC cell lines with MAPKAPK5-AS1 overexpression or knockdown. *p < 0.05.

**Additional file 5: Figure S3.** The level of MAPKAPK5-AS1 in subcutaneous tumor tissues. *p < 0.05.

**Additional file 6: Figure S4.** Prediction of targeted miRNAs for MAPKAPK5-AS1. (A) Subcellular fractionation assay was applied to determine MAPKAPK5-AS1 subcellular localization in Hep3B and Huh-7 cells. (B) The level of miR-1271–5p, miR-154-5p, and miR-450b-5p in HCCLM3 and MHCC97-H cells after MAPKAPK5-AS1 knockdown. (C) TCGA data accessed via ENCORI online platform showed the low miR-154-5p expression in HCC. *p < 0.05.

**Additional file 7: Figure S5.** MiR-154-5p mediates the function of MAPKAPK5-AS1. (A-C) MTT, colony formation and EdU assays were conducted to explore the effect of miR-154-5p mimics on cell proliferation enhanced by MAPKAPK5-AS1. (D) Flow cytometry showed the effect of miR-154-5p mimics on apoptosis inhibited by MAPKAPK5-AS1. (E-F) Transwell assay were performed to evaluate the effects of miR-154-5p mimics on the tumor cells abilities of migration and invasion enhanced by MAPKAPK5-AS1. (C-D) Expression of EMT markers was detected by western blotting to assess the effect of miR-154-5p mimics on EMT process promoted by MAPKAPK5-AS1. *p < 0.05.

**Additional file 8: Figure S6.** The level of PLAGL2 in HCC tissues and subcutaneous tumor tissues. (A-B) The effect of miR-154-5p on PLAGL2 mRNA level evaluated by qRT-PCR. (C) TCGA data accessed via ENCORI online platform showed the high PLAGL2 expression in HCC. (D) TCGA data accessed via ENCORI online platform showed the positive association between PLAGL2 and MAPKAPK5-AS1 in HCC. (E) The PLAGL2 expression in xenograft tumors.

**Additional file 9: Figure S7.** PLAGL2 mediates the oncogenic effect of MAPKAPK5-AS1. (A-C) MTT, colony formation and EdU assays showed the effect of PLAGL2 kncokdown on cell proliferation enhanced by MAPKAPK5-AS1. (D) Flow cytometry showed the effect of PLAGL2 kncokdown on cell apoptosis inhibited by MAPKAPK5-AS1. (E) Transwell assay showed the effects of PLAGL2 kncokdown on migration and invasion of the tumor cells promoted by MAPKAPK5-AS1. (F) Expression of EMT and EGFR/AKT pathway markers was detected by western blotting to assess the effect of PLAGL2 knockdown on EMT process and pathway activity promoted by MAPKAPK5-AS1. *p < 0.05.

**Additional file 10: Figure S8.** EGFR/AKT pathway mediates the oncogenic effect of MAPKAPK5-AS1. (A-B) MTT and EdU assays showed the the effect of MK2206, the inhibitor of AKT, on cell proliferation enhanced by MAPKAPK5-AS1. (C) Flow cytometry showed the effect of MK2206 on cell apoptosis inhibited by MAPKAPK5-AS1. (D) Transwell assay showed the effects of MK2206 on migration and invasion of the tumor cells promoted by MAPKAPK5-AS1. (E) Expression of EMT markers was detected by western blotting to assess the effect of MK2206 on EMT process promoted by MAPKAPK5-AS1. *p < 0.05.

## Data Availability

All data generated or analyzed during this study are included either in this article or in the supplementary information files.

## Abbreviations

HCC: Hepatocellular carcinoma
PLAGL2: Pleomorphic adenoma gene like-2
EGFR: Epidermal growth factor receptor
HIF: Hypoxia-inducible factor
EMT: Epithelial–mesenchymal transition
miRNA: MicroRNAs
3’UTR: 3′-Untranslated regions
ceRNA: Competitive endogenous RNA
TCGA: The cancer genome atlas
GEO: Gene expression omnibus
FBXW7: F-box and WD repeat domain containing 7
FOXA1: Forkhead box A1
RIP: RNA immunoprecipitation
ChIP: Chromatin immunoprecipitation
