# Supplementary material for: Long non-coding RNA MAPKAPK5-AS1/PLAGL2/HIF-1α signaling loop promotes hepatocellular carcinoma progression
Source: J Exp Clin Cancer Res. 2021 Feb 17;40:72. doi: 10.1186/s13046-021-01868-z (PMC7891009; doi:10.1186/s13046-021-01868-z)
Supplement: Supplementary file 1 — Additional file 1. [file 13046_2021_1868_MOESM1_ESM.docx]

**Additional File 1: Table S1 List of primers used in the study**

| **Gene** | **Primer sequences (5’-3’)** |
| --- | --- |
| MAPKAPK5-AS1-F | GGCGTCGTGAGGTATGGATGTTC |
| MAPKAPK5-AS1-R | GCTTGACCACTTCTCGGCTGTG |
| miR-154-5p-F | CGCGAATTCGCATCTAGGACCTCCATCAC |
| miR-154-5p-R | ACGGGATCCGAACCATCCCTTCACTTACC |
| PLAGL2-F | CCAGAGCAGAGACCATATAG |
| PLAGL2-R | AACATCTTATCACAGTACATACAC |
| GAPDH-F | CAAGGTCATCCATGACAACTTTG |
| GAPDH-R | TCCACCACCCTGTTGCTGTAG |
| U6-F | GCTTCGGCAGCACATATACTAAAAT |
| U6-R | CGCTTCACGAATTTGCGTGTCAT |
| MAPKAPK5-AS1 HRE-F | CACAGTGAAACCCCACCTCT |
| MAPKAPK5-AS1 HRE-R | TTGAGTCGGAGTCTGGCTTT |
