# Supplementary material for: Long non-coding RNA MAPKAPK5-AS1/PLAGL2/HIF-1α signaling loop promotes hepatocellular carcinoma progression
Source: J Exp Clin Cancer Res. 2021 Feb 17;40:72. doi: 10.1186/s13046-021-01868-z (PMC7891009; doi:10.1186/s13046-021-01868-z)
Supplement: Supplementary file 2 — Additional file 2. [file 13046_2021_1868_MOESM2_ESM.docx]

| **Antibody** | **Source** | **Catalog** |
| --- | --- | --- |
| E-Cadherin | Cell Signaling Technology | # 3195 |
| N-Cadherin | Cell Signaling Technology | #13116 |
| Vimentin | Cell Signaling Technology | #5741 |
| PLAGL2 | GeneTex | GTX32095 |
| EGF Receptor | Cell Signaling Technology | #4267 |
| Akt | Cell Signaling Technology | #4691 |
| Phospho-Akt (Thr308) | Cell Signaling Technology | #13038 |
| Phospho-Akt (Ser473) | Cell Signaling Technology | #4060 |
| Ki67 | Abcam | ab92742 |
| HIF-1α | Abcam | ab272040 |
| β-Actin | Cell Signaling Technology | #3700 |

**Additional File 2: Table S2 List of antibodies used in the study**
