# Supplementary figures and images for: Long non-coding RNA MAPKAPK5-AS1/PLAGL2/HIF-1α signaling loop promotes hepatocellular carcinoma progression
Source: J Exp Clin Cancer Res. 2021 Feb 17;40:72. doi: 10.1186/s13046-021-01868-z (PMC7891009; doi:10.1186/s13046-021-01868-z)

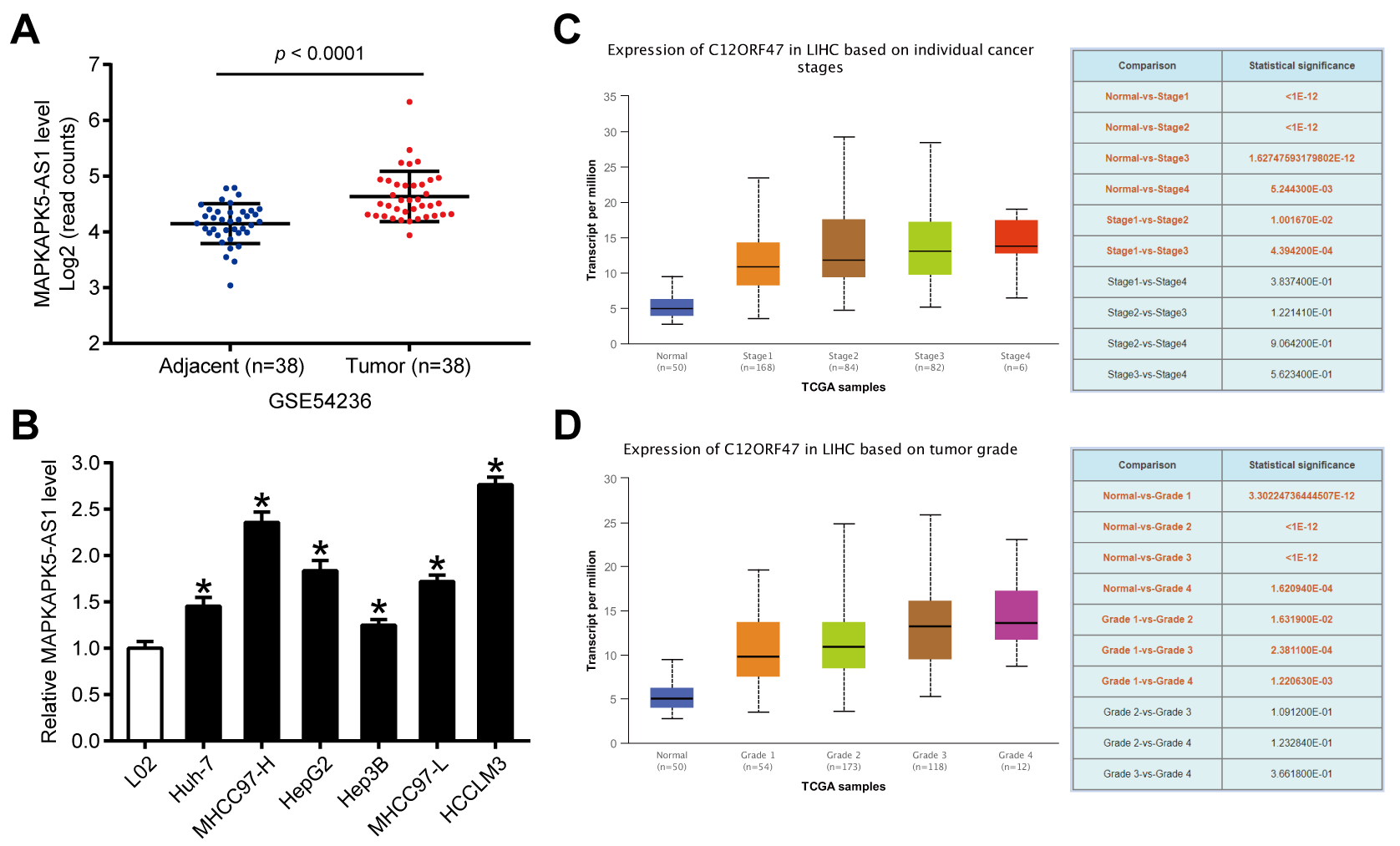

Supplement: Supplementary file 3 — Additional file 3: Figure S1. The expression and clinical significance of MAPKAPK5-AS1 in HCC. (A) GSE54236 dataset showed the high expression level of MAPKAPK5-AS1 in HCC tissues compared to normal tissues. (B) The level of MAPKAPK5-AS1 in human normal liver cell (L02) and HCC cell lines. (C) The association between MAPKAPK5-AS1 expression and pathological stage of HCC patients. (D) The association between MAPKAPK5-AS1 expression and tumor grade of HCC patients. *p < 0.05. [file 13046_2021_1868_MOESM3_ESM.tif]

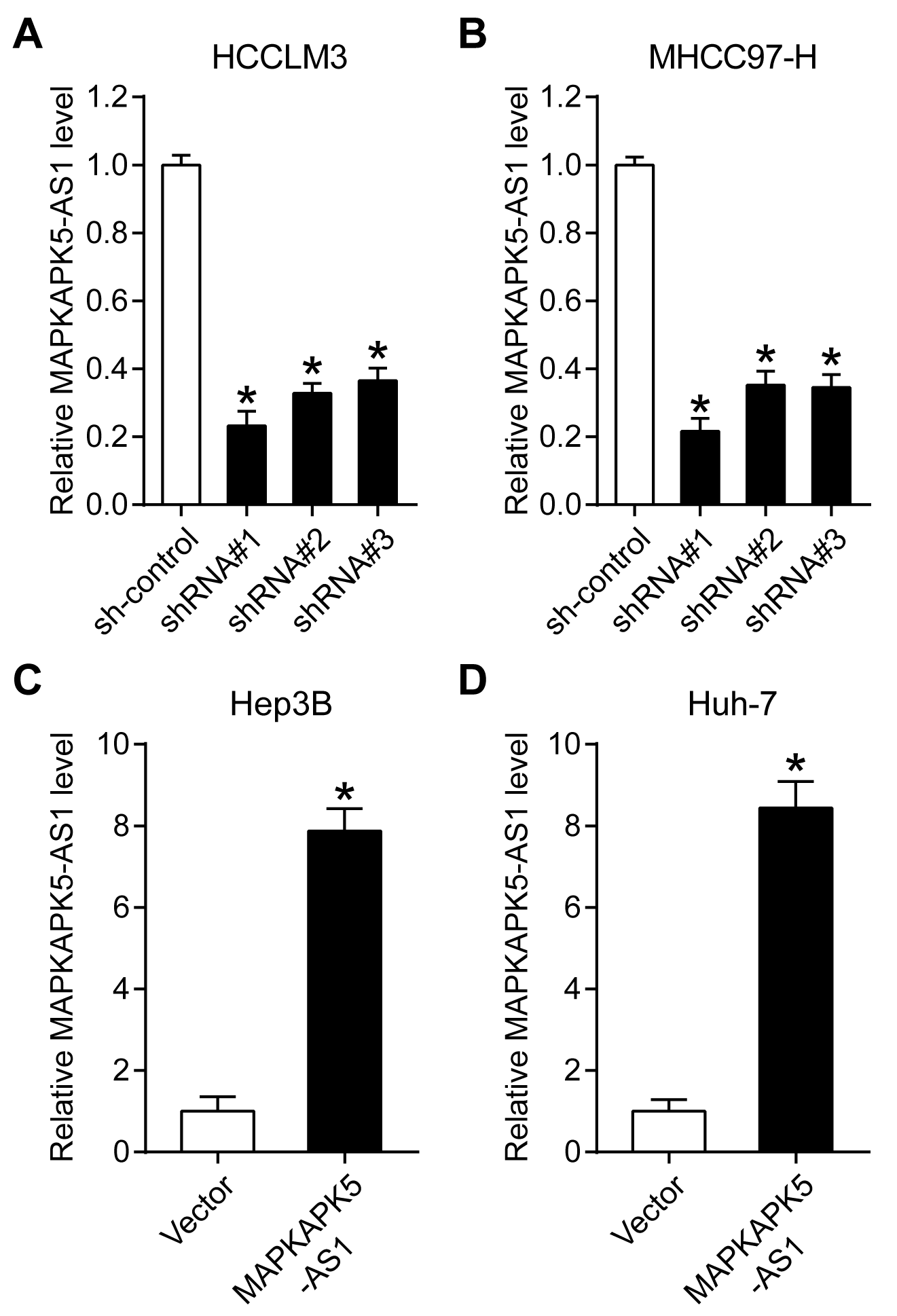

Supplement: Supplementary file 4 — Additional file 4: Figure S2. Transfection efficiency of these four HCC cell lines with MAPKAPK5-AS1 overexpression or knockdown. *p < 0.05. [file 13046_2021_1868_MOESM4_ESM.tif]

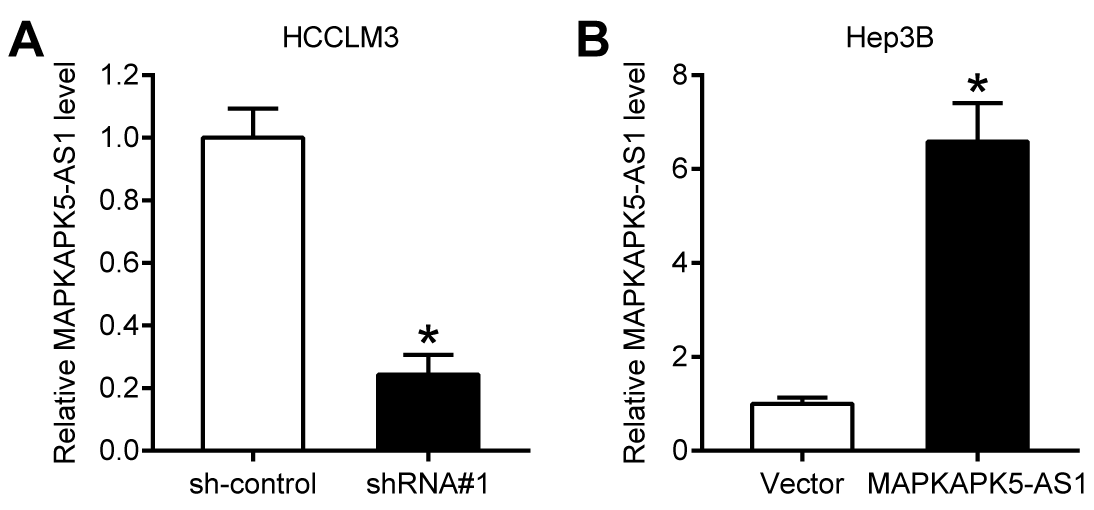

Supplement: Supplementary file 5 — Additional file 5: Figure S3. The level of MAPKAPK5-AS1 in subcutaneous tumor tissues. *p < 0.05. [file 13046_2021_1868_MOESM5_ESM.tif]

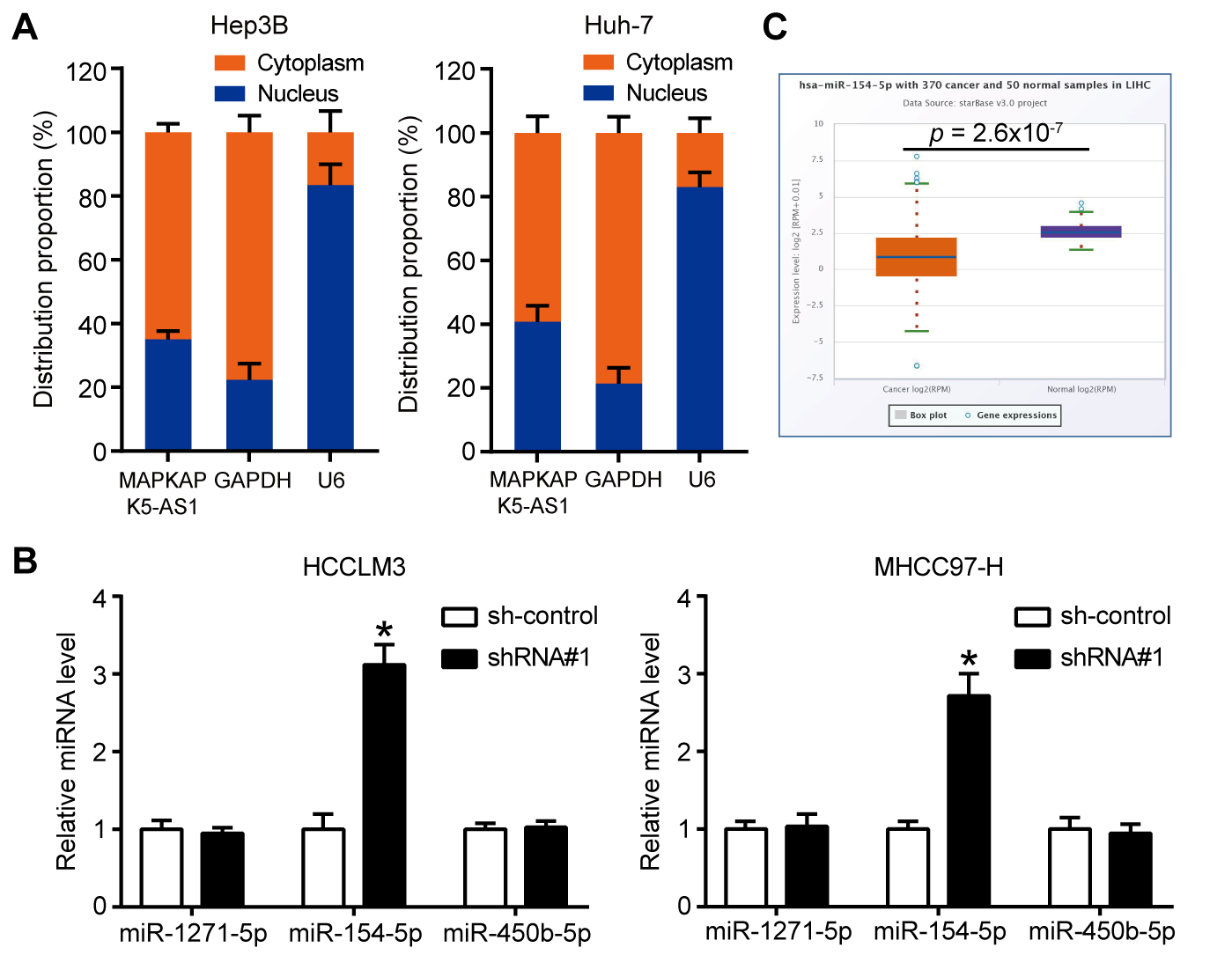

Supplement: Supplementary file 6 — Additional file 6: Figure S4. Prediction of targeted miRNAs for MAPKAPK5-AS1. (A) Subcellular fractionation assay was applied to determine MAPKAPK5-AS1 subcellular localization in Hep3B and Huh-7 cells. (B) The level of miR-1271–5p, miR-154-5p, and miR-450b-5p in HCCLM3 and MHCC97-H cells after MAPKAPK5-AS1 knockdown. (C) TCGA data accessed via ENCORI online platform showed the low miR-154-5p expression in HCC. *p < 0.05. [file 13046_2021_1868_MOESM6_ESM.tif]

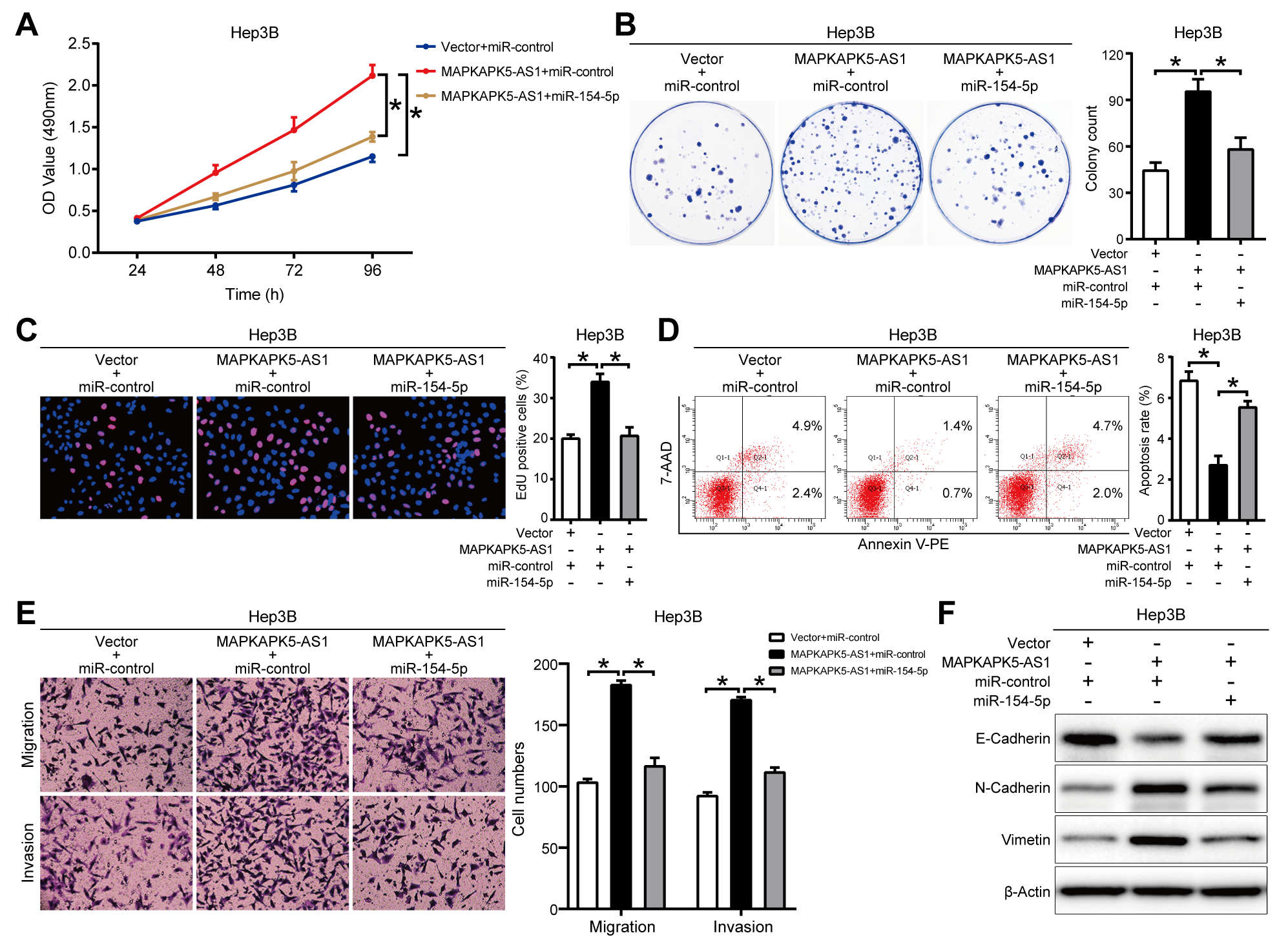

Supplement: Supplementary file 7 — Additional file 7: Figure S5. MiR-154-5p mediates the function of MAPKAPK5-AS1. (A-C) MTT, colony formation and EdU assays were conducted to explore the effect of miR-154-5p mimics on cell proliferation enhanced by MAPKAPK5-AS1. (D) Flow cytometry showed the effect of miR-154-5p mimics on apoptosis inhibited by MAPKAPK5-AS1. (E-F) Transwell assay were performed to evaluate the effects of miR-154-5p mimics on the tumor cells abilities of migration and invasion enhanced by MAPKAPK5-AS1. (C-D) Expression of EMT markers was detected by western blotting to assess the effect of miR-154-5p mimics on EMT process promoted by MAPKAPK5-AS1. *p < 0.05. [file 13046_2021_1868_MOESM7_ESM.tif]

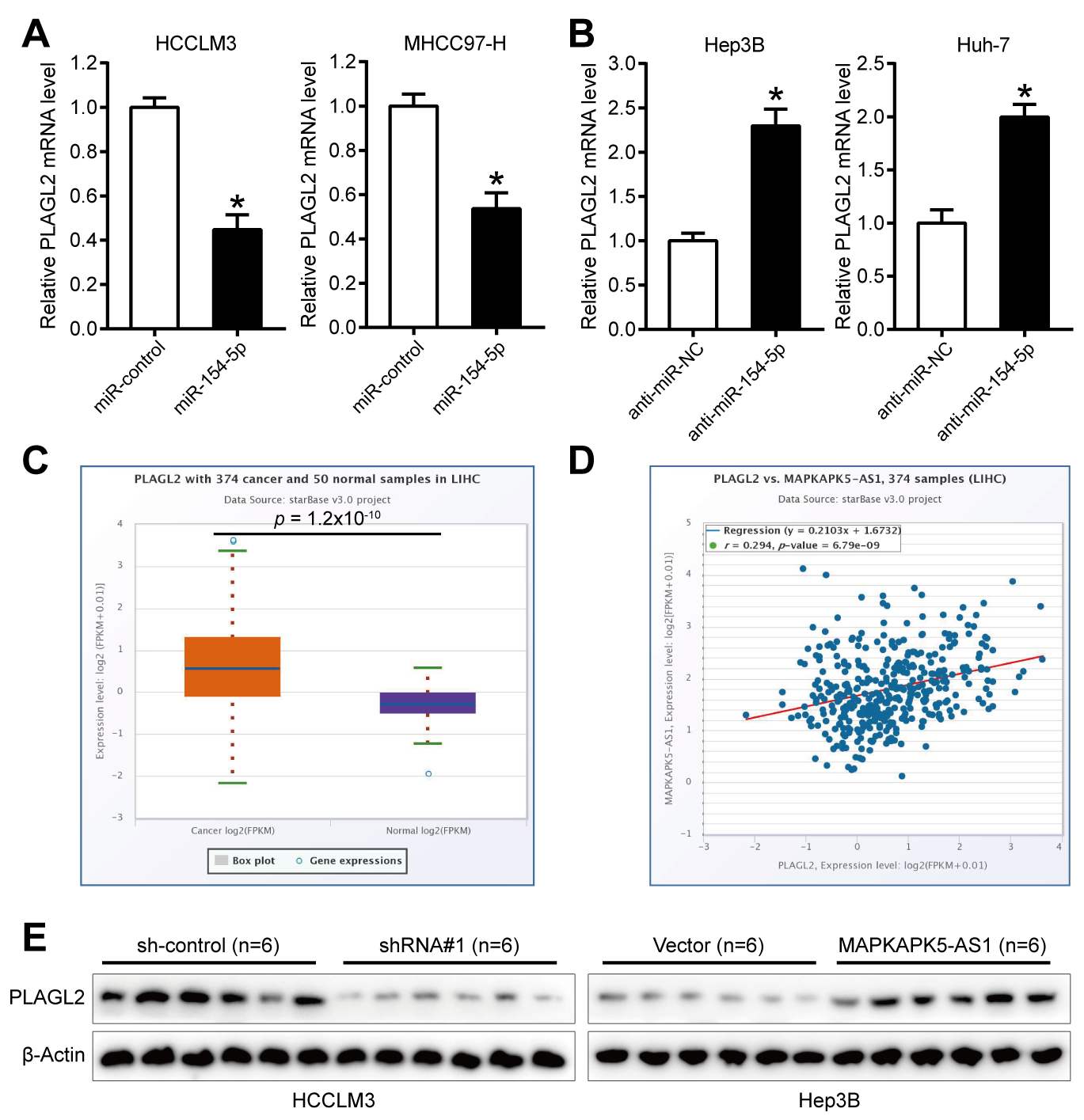

Supplement: Supplementary file 8 — Additional file 8: Figure S6. The level of PLAGL2 in HCC tissues and subcutaneous tumor tissues. (A-B) The effect of miR-154-5p on PLAGL2 mRNA level evaluated by qRT-PCR. (C) TCGA data accessed via ENCORI online platform showed the high PLAGL2 expression in HCC. (D) TCGA data accessed via ENCORI online platform showed the positive association between PLAGL2 and MAPKAPK5-AS1 in HCC. (E) The PLAGL2 expression in xenograft tumors. [file 13046_2021_1868_MOESM8_ESM.tif]

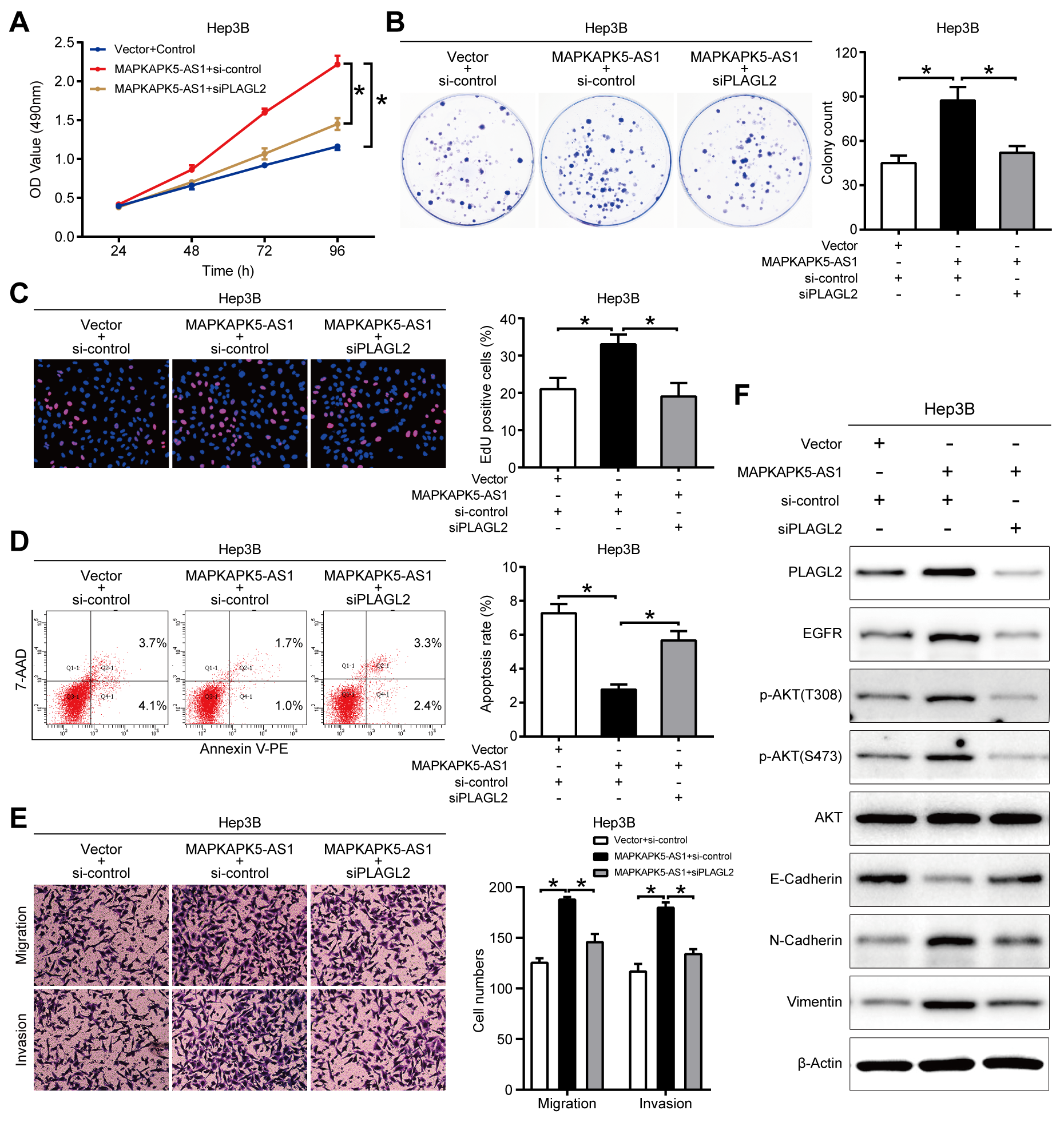

Supplement: Supplementary file 9 — Additional file 9: Figure S7. PLAGL2 mediates the oncogenic effect of MAPKAPK5-AS1. (A-C) MTT, colony formation and EdU assays showed the effect of PLAGL2 kncokdown on cell proliferation enhanced by MAPKAPK5-AS1. (D) Flow cytometry showed the effect of PLAGL2 kncokdown on cell apoptosis inhibited by MAPKAPK5-AS1. (E) Transwell assay showed the effects of PLAGL2 kncokdown on migration and invasion of the tumor cells promoted by MAPKAPK5-AS1. (F) Expression of EMT and EGFR/AKT pathway markers was detected by western blotting to assess the effect of PLAGL2 knockdown on EMT process and pathway activity promoted by MAPKAPK5-AS1. *p < 0.05. [file 13046_2021_1868_MOESM9_ESM.tif]

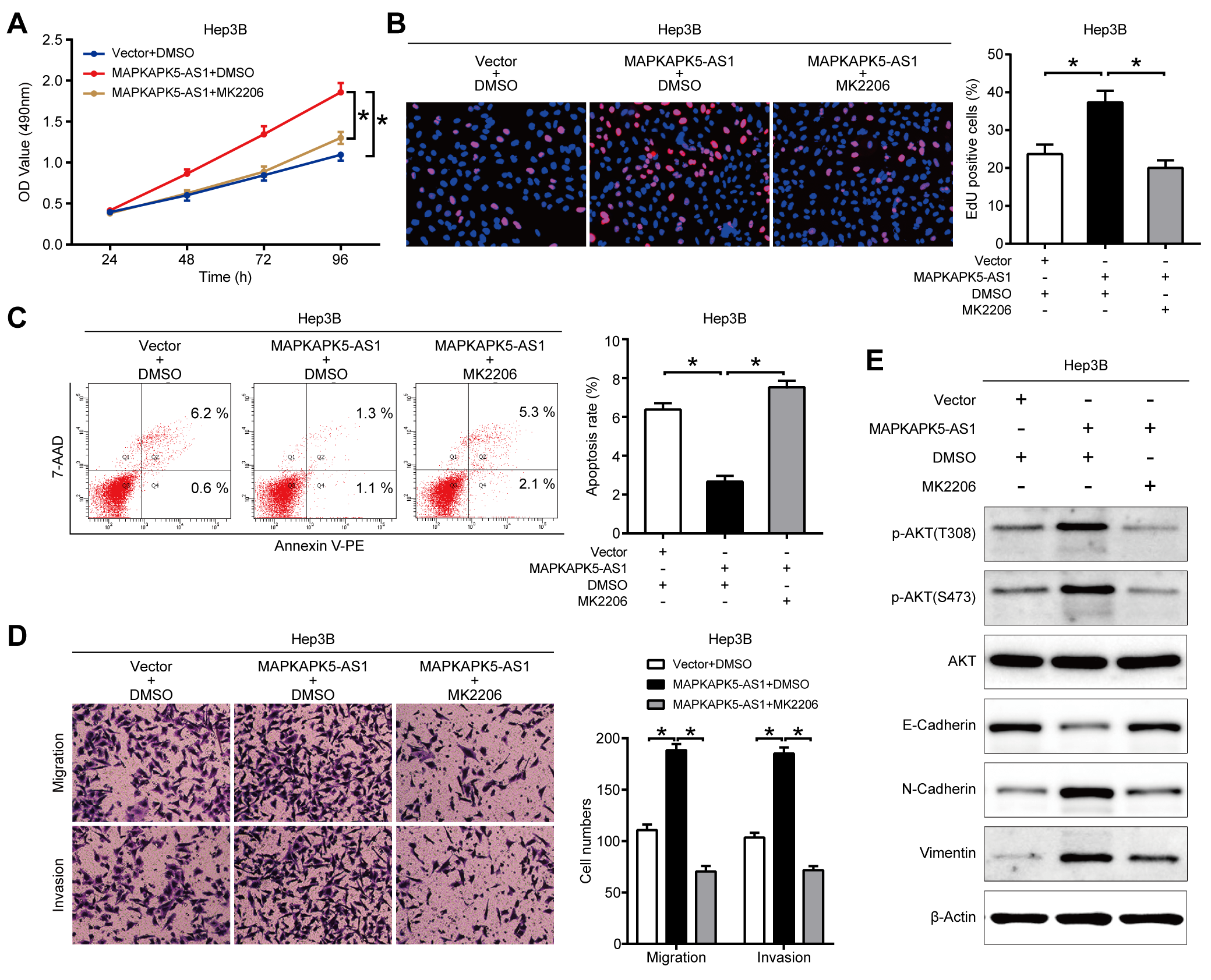

Supplement: Supplementary file 10 — Additional file 10: Figure S8. EGFR/AKT pathway mediates the oncogenic effect of MAPKAPK5-AS1. (A-B) MTT and EdU assays showed the the effect of MK2206, the inhibitor of AKT, on cell proliferation enhanced by MAPKAPK5-AS1. (C) Flow cytometry showed the effect of MK2206 on cell apoptosis inhibited by MAPKAPK5-AS1. (D) Transwell assay showed the effects of MK2206 on migration and invasion of the tumor cells promoted by MAPKAPK5-AS1. (E) Expression of EMT markers was detected by western blotting to assess the effect of MK2206 on EMT process promoted by MAPKAPK5-AS1. *p < 0.05. [file 13046_2021_1868_MOESM10_ESM.tif]
